# Telehealth to Address Preventable Maternal Deaths: A Call to Action

**DOI:** 10.1089/tmj.2024.0522

**Published:** 2024-12-06

**Authors:** Kelly A. Hirko, Ann Heler, Tamara Sampson

**Affiliations:** ^1^ Department of Epidemiology and Biostatistics, College of Human Medicine, Michigan State University, East Lansing, Michigan, USA.; ^2^ Free & Charitable Clinics of Michigan, Zeeland, Michigan, USA.; ^3^ Genesee Health System, Flint, Michigan, USA.

**Keywords:** telemedicine, telehealth, maternal health, postpartum care, equity

## Abstract

Over 80% of maternal deaths are preventable. Telehealth approaches can help address disparities by increasing access to quality maternal health care. In this position statement, we advocate for the utility of telehealth to address maternal mortality disparities, focusing specifically on the postpartum period, where most maternal deaths occur. Specifically, we describe how telehealth visits, mobile health applications, and wearable devices for remote patient monitoring can be used to promote the uptake of postpartum care and adherence to evidence-based treatment for the most common causes of maternal death (i.e., cardiovascular conditions and mental health-related conditions). We discuss challenges that must be overcome to ensure the broad and equitable reach of telehealth and identify specific action steps to address this pressing public health issue.

## Introduction

Maternal mortality rates in the United States (U.S.) are characterized by stark disparities for racial and ethnic minorities, rural residents, and people with lower socioeconomic status.^1^ The most striking and persistent disparity is for Black pregnant people, who are three times more likely to die from pregnancy-related complications than White people.^2^ Maternal mortality disparities represent an urgent public health crisis, particularly given that over 80% of U.S. maternal deaths have been deemed preventable.^3^

Inequitable access to quality maternity care is a key factor contributing to poor maternal health outcomes in medically underserved populations.^4^ Indeed, 36% of U.S. counties were designated as maternity care deserts, with no hospitals or birth centers offering obstetric care in 2022; the vast majority are located in rural regions.^5^ Access to maternity care has worsened in recent years,^5^ with additional restrictions expected as a result of recent legislative changes and workforce shortages.^6^ Social determinants of health also perpetuate maternal health disparities, contributing to the unequal presence of comorbidities and pregnancy complications that increase the risk for adverse maternal outcomes.^1^

A rising number of pregnant people in the U.S. have chronic health conditions that put them at higher risk of complications during pregnancy and beyond, such as chronic heart disease, mental illness, and substance use disorder.^7^ These preexisting conditions disproportionately burden marginalized communities, including those living in maternity care deserts.^7^ Taken together, rising inequities in maternal health care access and chronic health conditions among pregnant people emphasize the urgent need for effective strategies to prevent maternal deaths and address persistent maternal mortality inequities.

In this position statement, we advocate for the utility of telehealth approaches to address maternal mortality disparities perpetuated by inequitable access to quality health care and social determinants of health. Here, we describe telehealth as any health care delivery enhanced by telecommunications technologies, including audio-visual communication, mobile health applications, and wearable devices for remote patient monitoring. We focus specifically on the role of telehealth in the postpartum period for several reasons. First, the majority of maternal deaths in the U.S. occur after the baby is born.^3^ The most common underlying causes of maternal deaths, (i.e., cardiovascular conditions and mental health-related conditions), also disproportionately occur in the postpartum period.^3^ In addition, less attention has traditionally been paid to mothers during the “fourth trimester” where care generally shifts to infants. There is, however, a growing recognition of the importance of the postpartum period as a critical time of transition and opportunity to address health care and mental health needs. As such, the American College of Obstetricians and Gynecologists recently adopted guidelines to recommend ongoing comprehensive care after birth.^8^ Finally, inequities in postpartum care are extensive and compounded for people with multiple disadvantaged identities based on health insurance status, geography, race, and ethnicity.^9^

Thus, novel strategies to deliver quality maternal care during this postpartum period are urgently needed. While a comprehensive approach to improving the quality of care from preconception to postpartum is necessary to eliminate maternal mortality disparities, telehealth approaches may have a potentially more impactful role in this critical and often neglected postpartum period.

## Opportunities

There are numerous applications of telehealth to help address the wide-ranging health care inequities in the postpartum period. Here, we focus on telehealth approaches to improve access to postpartum care, describing specific examples of how telehealth can promote uptake of evidence-based treatment for the most common causes of maternal death, which disproportionately impact people who are medically underserved.

### INCREASE ACCESS TO POSTPARTUM CARE

Comprehensive care is critical to address physiological, social, and emotional changes in the postpartum period and to promote continuity of care for maternal morbidities that persist after birth.^9^ Telehealth approaches have been shown to increase access to maternity care, particularly for those in medically underserved communities, and to yield comparable or even improved outcomes compared with in-person care.^10^ Results from a recent systematic review of the literature suggest the utility of virtual follow-up visits to improve continuity of care in the postpartum period,^11^ overcoming barriers to in-person care by reducing transportation challenges, and time concerns related to employment and childcare responsibilities.^12^ Moreover, postpartum care models that include the provision of virtual visits offer flexibility, supporting a patient-centered approach that can help increase uptake and patient satisfaction.^13^

Telehealth approaches can also help to overcome access barriers related to workforce shortages in maternity care deserts by bridging the divide between maternity care providers and specialists. For example, telehealth can support existing hub-and-spoke models of care delivery in which obstetricians and maternal-fetal medicine specialty care providers from larger health care systems (the “hubs”) support maternity and/or primary care providers in smaller health care systems (the “spokes”). This collaborative web-based model helps to fill gaps throughout the maternity care continuum while also building local capacity. An example of this distance-learning model approach is the Pregnancy Care Extension for Community Health Outcomes program, which leverages videoconferences to train nurses, advanced practice providers, and physicians in medically underserved communities to disseminate and implement best practices in maternity and postpartum care.^14^ Given trends of declining access to maternity care in recent years,^5^ it will also be important to consider integrated care delivery models, enlisting other partners to provide emotional and practical support during the postpartum period, including doulas, midwives, and patient navigators to improve access to and retention in postpartum care.^15^

### IMPROVE UPTAKE OF EVIDENCE-BASED POSTPARTUM CARE

Postpartum follow-up care is critical to the prevention and treatment of the underlying causes of maternal mortality, which often arise during pregnancy. Quality comprehensive postpartum care entails careful screening, monitoring, and referral to evidence-based prevention and treatment, which can all be facilitated using telehealth approaches. In this paper, we focus on how telehealth can be used to address disparities resulting from the most common causes of maternal death during the postpartum period—cardiovascular conditions and mental health-related conditions.

Telehealth approaches in the postpartum period are well suited to identify those with high-risk and deliver evidence-based cardiovascular prevention and treatment programs. For example, text-based models using secure health messaging portals and patient-reported outcome assessments can help identify those with high-risk who may need additional follow-up and monitoring during the postpartum period.^11^ Text-based communication strategies have already proven effective for managing postpartum blood pressure in women with hypertensive disease of pregnancy, while also reducing emergency department visits and hospital readmissions related to hypertension.^16^ For those with high-risk conditions (e.g., hypertension), digital health approaches such as remote patient monitoring using a tablet with embedded devices can monitor blood pressure, effectively engaging people in their care. Importantly, these approaches have demonstrated feasibility and acceptability in diverse populations, reducing disparities in blood pressure monitoring during the postpartum period.^17^ Telehealth approaches can also be used to facilitate the delivery of evidence-based cardiovascular disease prevention efforts. For example, telehealth approaches work well in reinforcing health behaviors associated with prevention of cardiovascular conditions, such as smoking cessation.^10^ Moreover, telehealth can support enhanced collaboration and coordination of care between obstetrician-gynecologists and cardiologists to improve outcomes for people at high-risk of adverse cardiovascular outcomes.^18^

Postpartum depression and substance use disorder (SUD) are high-burden conditions, with effective behavioral therapies available that can be delivered remotely via telehealth approaches. For example, text-based reminders incorporating patient-reported outcome measures can be used to screen for postpartum depression and SUD, and cognitive behavioral therapy can be delivered via virtual follow-up visits. Notably, telehealth interventions (both web-based and telephone-based behavioral therapy) have been shown to be acceptable, feasible, and effective for preventing and mitigating the symptoms of postpartum depression and its downstream impacts.^19^ There is also a rising interest in using mobile health technologies to help people self-manage postpartum depression symptoms, but more evidence on effectiveness of these mobile apps is needed.

## Challenges

While telehealth approaches have enormous potential to overcome barriers to postpartum care, challenges to broad and equitable implementation exist. Optimizing telehealth approaches for postpartum care must ensure accessible and acceptable delivery strategies. Unfortunately, the current reach of telehealth is limited by geographic inequities in broadband internet access, and areas with low broadband access also tend to have limited access to maternity care.^20^ Issues of digital literacy are also pronounced, leading to mistrust and privacy concerns that can impede the uptake of telehealth-based care delivery. Moreover, the integration and implementation of telehealth into health care systems requires substantial upfront costs, including provision of necessary hardware, software, and secure and reliable internet connectivity for both providers and those receiving care.

Challenges related to payment parity, including the generally lower reimbursement for telehealth services versus in-person, may discourage providers and health care systems from implementing these programs. Provider payment parity mandates are needed to reassure providers that telehealth services will be reimbursed. In additon, there is a substantial gap in knowledge, with limited rigorous evidence supporting telehealth interventions in postpartum care.^10^ While technology-enhanced health care delivery has rapidly expanded in recent years, the development of evidence-based postpartum interventions that can be delivered remotely lag behind.^10^ As such, there is an urgent need for robust research on telehealth approaches in maternal health to guide practice and ensure that burgeoning technologies can be utilized while minimizing risks.

Finally, it is important to note that telehealth approaches cannot necessarily overcome all barriers to accessing postpartum care, including poverty, implicit bias, stigma, structural inequities, and cost of care for the uninsured/underinsured. Multifaceted approaches are undoubtedly needed to comprehensively address persistent maternal mortality disparities.

## Call to Action: Next Steps

The roadmap for equitably moving telehealth services into postpartum care models requires much work. We advocate for the following steps to address this pressing public health crisis.

1. Develop training opportunities for people to provide and receive virtual postpartum care, ensuring everyone can benefit from telehealth. These programs should promote patient-centered technical support to address difficulties downloading software, navigating telehealth visits, and addressing privacy concerns. Efforts to simplify and consolidate telehealth platforms are also needed. Investing in training programs for nurse midwives and doulas that can be delivered remotely can also help fill the gaps in postpartum care in maternity care deserts.
2. Identify strategies for cost-effective implementation of telehealth services in the postpartum period. This includes research and rigorously evaluated telehealth technologies focused on clinical value and return on investment to ensure equitable implementation opportunities.
3. Advocate for policy changes to ensure continued coverage postpartum, payment parity for telehealth services, and expanded coverage for remote patient monitoring. Ensure that policies support audio-only visits while continuing efforts to expand access to affordable broadband internet services. Promote expanded telehealth-eligible delivery of Medicaid services in the postpartum period, eliminating distance requirements and restrictions.
4. Leverage technology approaches to deliver and support existing evidence-based postpartum care interventions (e.g., State maternal infant health programs). Continue to focus on developing evidence-based postpartum care interventions that can be delivered remotely to increase the reach of these services.
5. Engage communities of people who are medically underserved in efforts to develop and implement telehealth-based postpartum care models. Ensure that the interventions and implementation strategies for addressing health care needs in the postpartum period are culturally informed and tailored to the community.

## Conclusions

There is an urgent need to expand the reach of evidence-based postpartum care to prevent unnecessary maternal deaths and address persistent maternal health inequities. Telehealth approaches can be leveraged to overcome these gaps in care. Challenges related to broadband internet access, digital literacy, payment parity, and cost-effective implementation will need to be addressed to ensure broad and equitable reach of these services. A health equity lens is recommended, with interventions and implementation strategies that are adapted to meet the needs of disproportionately burdened populations. Overcoming these challenges can help to address a broad range of health inequities for people in medically underserved communities around the globe.
